# Scutellarin regulates the Notch pathway and affects the migration and morphological transformation of activated microglia in experimentally induced cerebral ischemia in rats and in activated BV-2 microglia

**DOI:** 10.1186/s12974-014-0226-z

**Published:** 2015-01-20

**Authors:** Yun Yuan, Parakalan Rangarajan, Enci Mary Kan, Yajun Wu, Chunyun Wu, Eng-Ang Ling

**Affiliations:** Department of Anatomy and Histology/Embryology, Faculty of Basic Medical Sciences, Kunming Medical University, 1168 West Chunrong Road, Kunming, 650500 PR China; Department of Anatomy, Yong Loo Lin School of Medicine, National University of Singapore, 4 Medical Drive, MD10, Singapore, 117597 Singapore; Defense Medical and Environmental Research Institute, DSO National Laboratories, 27 Medical Drive, Singapore, 117510 Singapore

**Keywords:** scutellarin, Notch pathway, activated microglia, cytoskeleton, migration, cerebral ischemia

## Abstract

**Background:**

Activated microglial cells release an excess of inflammatory mediators after an ischemic stroke. We reported previously that scutellarin effectively suppressed the inflammatory response induced by activated microglia in rats subjected to middle cerebral artery occlusion (MCAO); however, the mechanism *via* which scutellarin exerts its effects on microglial activation has not been explored. This study aimed to elucidate if scutellarin can regulate the Notch pathway that is linked to microglia activation in MCAO rat, and in lipopolysaccharide (LPS)-induced BV-2 microglia. Along with this, we also investigated some characteristic behavioral responses of activated microglia.

**Methods:**

Expression of various members of the Notch pathway, including Notch-1, intracellular Notch receptor domain (NICD), recombining binding protein suppressor of hairless (RBP-JK) and transcription factor hairy and enhancer of split-1 (Hes-1) in activated microglia was assessed by immunofluorescence staining and western blot after experimental MCAO and *in vitro* LPS activation. The effect of scutellarin on migration of microglia was determined by the transwell chamber assay as well as expression of monocyte chemoattractant protein-1 (MCP-1). The morphological change of microglia induced by scutellarin was detected by F-actin staining and electron microscopy.

**Results:**

Scutellarin markedly attenuated the expression of NF-κB, Notch-1, NICD, RBP-JK and Hes-1 both *in vivo* and in activated microglia. It decreased the expression of MCP-1 and microglial migration, but increased the ability of microglia adhesion. Scutellarin also altered the phenotype of microglia by causing rearrangement or reorganization of its cytoskeleton.

**Conclusions:**

The results suggest that scutellarin regulates the activation of microglia *via* the Notch pathway and concurrently induces morphological and functional changes in activated microglia.

**Electronic supplementary material:**

The online version of this article (doi:10.1186/s12974-014-0226-z) contains supplementary material, which is available to authorized users.

## Introduction

Stroke is a cerebrovascular neuropathology that causes severe neurological deficits leading to long-term disability [1]. Stroke is either caused by ischemia, which is triggered by the blockade of blood vessels supplying the brain [2] or hemorrhage, which is due to bleeding in the brain parenchyma [3]. In the event of a stroke, microglia, which are the innate immune cells of the central nervous system (CNS), are activated [4]. These activated microglia then migrate to the infarct area to perform phagocytotic clearance of cellular debris [5], thus offering neuroprotection following ischemia. On the other hand, the prolonged and non-specific inflammatory reaction of activated microglia may cause injury to healthy neurons, resulting in progressive neuronal damage [6]. Microglia are thought to contribute to neuronal damage *via* release of excessive proinflammatory cytokines and/or cytotoxic factors, such as nitric oxide (NO), tumor necrosis factor-α (TNF-α), interleukin-1β (IL-1β), and reactive oxygen species (ROS). Activation of microglia following ischemia has been shown to occur over a period of several months after the initial attack [7]. Therefore, this therapeutic window may be utilized to moderate microglial activation. It is well documented that microglia undergo a series of morphological transformation and functional alterations, including proliferation and migration when activated by an injurious or infectious stimulus. Therefore, it is essential to understand and design ways to modulate these functional aspects of activated microglia, so as to ameliorate neuronal damage caused by microglia-mediated neuroinflammation after stroke.

In the search for a potential drug that might help reduce microglia-mediated neuroinflammation, there has been increasing interest on scutellarin (4,5,6-trihydroxyflavone-7-glucuronide), a Chinese herbal compound reported to possess antioxidant properties [8,9]. Scutellarin has been shown to attenuate microglial inflammatory response [10]. In addition to its antioxidant and anti-inflammatory properties, scutellarin has been demonstrated to have anti-apoptotic properties in animal models of ischemic stroke [11]. However, the direct molecular targets of this drug in activated microglia have not been elucidated.

The Notch signaling pathway has been shown to control many functions in various types of tissues, both in healthy as well as in pathological conditions. In the developing CNS, the interaction between Notch and its ligands Delta-1 and Jagged-1 has been reported to trigger off a cascade of biochemical events that eventually regulates neuronal differentiation and gliogenesis [12]. In addition, the Notch pathway is also implicated in the progression of CNS pathologies such as Alzheimer’s disease (AD) and ischemic stroke [13]. Our previous report showed that Notch-1 and its ligands Jagged-1 and Delta-1 are expressed in the amoeboid microglia in the early postnatal rat brain, and regulate the microglial expression of pro-inflammatory cytokines when activated with lipopolysaccharide (LPS) [14]. Recently, we have also shown a vital role for the Notch pathway in activated microglia in response to hypoxic brain injury through its transactivation of NFκB and subsequent cytokine release [15]. Remarkably, inhibition of γ-secretase, an enzyme that cleaves Notch-1 into notch intracellular domain (NICD), led to suppression of microglial activation following hypoxia [15]. In addition, several studies have reported a central role for the Notch signaling pathway in other cellular mechanisms such as migration [16,17], proliferation [18,19], and phagocytosis [20]. In order to modulate microglial activation, it would be desirable to unravel the role of Notch and its downstream signaling molecules involved in microglial proliferation, migration, and phagocytosis.

In light of the above, we aimed to investigate if scutellarin is able to affect the functions of activated microglia *via* regulation of the Notch pathway, through the use of the experimental middle cerebral artery occlusion (MCAO) model of ischemic stroke and *in vitro* LPS activation of BV-2 microglial cells. The study aims to provide a deeper understanding of the molecular mechanism *via* which scutellarin acts upon the Notch-NFκB pathway in activated microglia, so as to open up the possibility of these pathways to be targeted to attenuate microglia-mediated neuroinflammation and to aid in the functional recovery of brain tissue following ischemic stroke.

## Methods

### Animals and injection of scutellarin

A total of 108 adult male Sprague-Dawley rats weighing 250 to 280 g were obtained from the Experimental Animal Center of Kunming Medical University. All animals were cared for according to National Institutes of Health Guide for the Care and Use of Laboratory Animals. All experimental protocols and the use of animals were approved by Kunming Medical University. All efforts were made to minimize the number and suffering of the rats used. The animals were randomly divided into sham-operated + saline (sham), MCAO + saline (MCAO), MCAO + scutellarin low dose (50 mg/kg) (S_L_), and MCAO + scutellarin high dose (100 mg/kg) (S_H_) groups (Table 1).

**Table 1:** **Surgical procedures and number of rats used in various treatments**

	**Sham-operated + saline group (sham)**	**MCAO + saline group (MCAO)**	**MCAO + scutellarin low dose group (S**_**L**_ **)**	**MCAO + scutellarin high dose group (S**_**H**_ **)**
**Double inmunofluorescence**	**n = 9**	**n = 15**	**n = 15**	**n = 15**
**Western blot**	**n = 9**	**n = 15**	**n = 15**	**n = 15**
**Total**	**n = 18**	**n = 30**	**n = 30**	**n = 30**

MCAO, middle cerebral artery occlusion.

The animals were anesthetized with an intraperitoneal injection of sodium pentobarbital (50 mg/kg). Briefly, a circular aperture 3 mm in diameter was made in the right parietal bone with a dental drill, and the main trunk of the middle cerebral artery (MCA) was exposed and cauterized [21]. In the sham-operated rats, the same surgical procedure was followed, but the MCA was not cauterized. The rats in the respective drug groups were given an intraperitoneal injection of scutellarin (50 mg/kg (low dose) or 100 mg/kg (high dose) dissolved in saline; purity 99%, Cat. No. 131021, Shanghai Winherb Medical Technology, China) at 2 h before and at 12, 24, 36, 48, and 60 h after MCAO; rats were sacrificed at 1, 3 and 7 d after MCAO.

### Dose-dependency of scutellarin effects in BV-2 cells

BV-2 cells were pretreated with different dosages of scutellarin (0.54 mM, 0.1 mM, 0.02 mM) for 1 h followed by LPS (1 μg/ml) incubation for 3 h. The protein expression of inducible nitric oxide synthase (iNOS) and TNF-α was then detected by western blot analysis. The information of both antibodies used is given in Table 2. Both iNOS and TNF-α were selected for this purpose because it is well documented that they are typical biomarkers for activated microglia, which has been demonstrated previously by us [22], as well as by others. It was found that scutellarin decreased the expression level of both proteins in a dose-dependency manner with the effect of 0.54 mM scutellarin being most effective (Additional file 1: Figure S1). Therefore, we have used 0.54 mM dosage for all *in vitro* experiments in this study. Along with this, we have also evaluated scutellarin at different dosages on Notch expression in LPS-activated BV-2 cells, and this has yielded a similar dose-dependency effect.

**Table 2:** **Antibodies used for western blotting and staining**

**Antibody**	**Host**	**Source**	**Catalog number**
NFκB	Rabbit polyclonal	Santa Cruz Biotechnology, CA, USA	sc-109
Notch-1	Rabbit polyclonal	Santa Cruz Biotechnology, CA, USA	sc-6014-R
NICD	Rabbit polyclonal	Merck KGaA, Darmstadt, Germany	07-1232
RBP-Jk	Rabbit polyclonal	Santa Cruz Biotechnology, CA, USA	sc-28713
Hes-1	Rabbit polyclonal	Santa Cruz Biotechnology, CA, USA	sc-25392
MCP-1	Rabbit polyclonal	Santa Cruz Biotechnology, CA, USA	sc-28879
Integrin β2	Rabbit polyclonal	Santa Cruz Biotechnology, CA, USA	sc-28661
RhoA	Mouse monoclonal	Santa Cruz Biotechnology, CA, USA	sc- 418
Rac1	Mouse monoclonal	Millipore Corporation, CA, USA	2328346
iNOS	Mouse monoclonal	BD Pharmingen San Jose, CA USA	610432
TNF-α	Rabbit polyclonal	Chemicon, Temecula, CA, USA	AB2148P
β-actin	Mouse monoclonal	Sigma-Aldrich, MO, USA	A-5441

### Double immunofluorescence labeling in the cerebrum and BV-2 microglial cells

A total of 15 rats of various experimental groups were used for double immunofluorescence labeling: 1, 3, and 7 days (n = 5 at each time point) along with 9 rats for sham (n = 3 at each of the 3 time points). Following deep anesthesia with 6% sodium pentobarbital, the rats were sacrificed by perfusion with 2% paraformaldehyde in 0.1 M phosphate buffer. The brain was removed and paraffin embedded. Coronal sections of 7 μm thickness were cut on a microtome (Model: 2165; Leica, Bensheim, Germany). For blocking of nonspecific binding proteins, tissue sections were incubated in 5% normal goat serum diluted in phosphate-buffered saline (PBS) for 1 h at room temperature (22 to 24°C). The sections were then incubated in a humidified chamber with the following primary antibodies: monocyte chemoattractant protein-1, MCP-1 (rabbit polyclonal IgG 1:100) (Santa Cruz Biotechnology, Cat. No. sc-28879); Notch-1 (rabbit polyclonal IgG 1:100) (Santa Cruz Biotechnology, Cat. No. sc- 6014-R); intracellular Notch receptor domain, NICD (Rabbit polyclonal IgG 1:200) (Merck KGaA, Cat. No. 07-1232); recombining binding protein suppressor of hairless, RBP-JK (rabbit polyclonal IgG 1:100) (Santa Cruz Biotechnology, Cat. No. sc- 28713); transcription factor hairy and enhancer of split-1, Hes-1 (rabbit polyclonal IgG 1:100) (Santa Cruz Biotechnology, Cat. No. sc- 25392); and nuclear factor kappa-light-chain-enhancer of activated B cells, NFκB (rabbit polyclonal IgG, 1:100) (Santa Cruz Biotechnology, Cat. No. sc-109) diluted in PBS overnight at 4°C. Following washing in PBS, sections were incubated, with the respective fluorescent secondary antibodies: Cy3-conjugated secondary antibody and FITC-conjugated lectin (*Lycopersicon esculentum*), which labels both microglia and blood vessel endothelial cells for 1 h at room temperature. After three rinses with PBS, the sections were mounted with a fluorescent mounting medium containing 4′,6-diamidino-2-phenylindole (DAPI) (Sigma, USA; Cat. No. F6057). Colocalization was observed by confocal microscopy (Fluoview 1000, Olympus Company Pte. Ltd., Tokyo, Japan). The details of the antibodies used are given in Table 2.

BV-2 microglial cells were cultured in Dulbecco’s modified Eagle’s medium (DMEM), supplemented with 10% fetal calf serum (FCS) at 37°C in a humidified incubator under 5% CO_2_. The cells were divided into control, LPS-induced and LPS + scutellarin groups. The cells were pretreated with scutellarin (0.54 mM) for 1 h at 37°C in a humidified incubator under 5% CO_2_. This dosage used was based on the cell viability assay done previously by us [22], as well as on the dose-dependency results (see Additional file 1: Figure S1). After incubation, the medium was discarded and the cells were washed with PBS, and then incubated with LPS (1 μg/ml, Sigma-Aldrich, MO, USA) for 3 h. The culture medium was replaced with basic DMEM before treatment. For controls, the medium was replaced with basic DMEM. The cells were then fixed with 4% paraformaldehyde in 0.1 M PBS for 20 min. Following rinsing with PBS, the cover slips with adherent cells were used for immunofluorescence staining. In each group, BV-2 microglial cells were incubated with the primary antibodies as described above overnight at 4°C. Subsequently, the cells were incubated with FITC/Cy3-conjugated secondary antibodies for 1 h at room temperature. After washing, the cover slips were mounted using a fluorescent mounting medium with DAPI. All images were captured using a confocal microscope.

### Western blotting analysis for middle cerebral artery occlusion tissues and BV-2 microglial cells

A total of 54 rats were used for western blotting analysis. The sham-operated rats (n = 9, 3 per time point), and MCAO rats given saline, and scutellarin (two different dosages, S_L_ and S_H_) injections were sacrificed at 1 (n = 5 for each group), 3 (n = 5 for each group) and 7 days (n = 5 for each group), respectively. The ischemic cortex and corresponding area in the controls derived from each group were frozen in liquid nitrogen and stored at -80°C. Tissue samples from various groups were homogenized with protein extraction reagent (Pierce, IL, USA) containing protease inhibitors. For BV-2 microglial cells of different groups (the control, LPS-induced and LPS + scutellarin), the cells were lysed with lysis buffer, mechanically scraped off with a rubber scraper and centrifuged at 13,000 rpm for 25 min. Protein concentrations of both tissues and BV-2 microglial cells were determined by using a protein assay kit (Bio-Rad, Hercules, CA, USA; Cat. No. 500-0002). Samples of supernatants containing 50 μg protein of tissue lysate or 40 μg protein of BV-2 microglial cells were loaded and heated to 95°C for 5 min, and then separated by sodium dodecyl sulfate-poly-acrylamide gel electrophoresis in 10% and 12% gels respectively, in a Mini-Protein II apparatus (Bio-Rad, CA, USA). Protein bands were electroblotted onto polyvinylindene difluoride (PVDF) membrane and blocked with non-fat dried milk for 1 h. The membranes were incubated with MCP-1 (rabbit polyclonal IgG 1:1000) (Santa Cruz Biotechnology; Cat. No. sc-28879), Notch-1 (rabbit polyclonal IgG 1:1500) (Santa Cruz Biotechnology, Cat. No. sc- 6014-R), NICD (Rabbit polyclonal IgG 1:4000) (Merck KGaA, Cat. No. 07-1232), RBP-JK (rabbit polyclonal IgG 1:500) (Santa Cruz Biotechnology, Cat. No. sc- 28713), Hes-1 (rabbit polyclonal IgG 1:300) (Santa Cruz Biotechnology, Cat. No. sc- 25392) and β-actin (mouse monoclonal IgG 1:10000) (Sigma; Cat. No. A5441) primary antibodies (Table 2) diluted in Tris-Buffered Saline-0.1% Tween (TBST) overnight at 4°C. They were then incubated with the secondary antibodies, either with horseradish peroxidase conjugated anti-rabbit IgG (Thermo Scientific; Cat. No. 31460) or anti-mouse IgG (Thermo Scientific; Cat. No. 31430). Protein was detected by a chemiluminescence kit (GE Healthcare UK Limited, Bucks, UK) following the manufacturer’s instructions and developed on film. The band intensity was quantified in Image J software (National Institutes of Health, NIH, USA). All experiments were repeated at least in triplicate. In addition, nuclear protein of BV-2 microglial cells was extracted to detect the effect of scutellarin on NICD transferring to nucleus. Nuclear protein extraction was performed by using the NE-PER Nuclear and Cytoplasmic Extraction Kit (Thermo Scientific, MA, USA. Cat. No. 78833) following the manufacturer’s instructions and the amount of protein was determined by using a protein assay kit (Bio-Rad, Hercules, CA, USA; Cat. No. 500-0002).

### Cell migration assay

The effect of scutellarin on BV-2 microglial cell migration was assessed using Transwell™ permeable support polycarbonate membrane inserts (8.0 μm pore size; Costar, USA), placed in a 24-well plate. After treatment with scutellarin at different concentrations (0.54 mM, 0.27 mM and 0.11 mM) for 3 h, the cells were washed with PBS, trypsinized, and resuspended in basic medium. The 200-μl cell suspension without FCS (3 × 10^4^ cells) was seeded into the inside of the insert, and 600 μl DMEM with 20% FCS was added into each well of the 24-well plate. After incubation at 37°C with 5% CO_2_ for 20 h, the medium was discarded and the cells remaining inside the insert were carefully removed. Cells were then fixed with 100% methanol for 15 min at room temperature and were stained with 0.5% of crystal violet at room temperature for 30 min. The cells were rinsed with distilled water until excess dye was removed. The images of migrated cells on the lower surface of the insert were captured at a magnification of 120x. Cells in five visual fields were counted on each insert using Image J software. Results are expressed as mean (± SD) of the number of cells per visual field.

### Cell adhesion assay

BV-2 microglial cells were plated in 6-well plates at a density of 3 × 10^5^ cells/well for 24 h following treatment with scutellarin at different concentrations (0.54 mM, 0.27 mM and 0.11 mM) for 3 h. The cells were then trypsinized and shaken at 80 rpm for 3 min. The images of cells left on the surface of the plate were captured at a magnification of 200x. Five visual fields were randomly selected and the cells in these visual fields were counted using Image J software. The percentage of cells that remained adherent to the wells was calculated as follows:

$$ \frac{\mathrm{sum}\;\mathrm{of}\;\mathrm{number}\;\mathrm{of}\;\mathrm{cells}\kern0.24em \mathrm{in}\ \mathrm{five}\;\mathrm{visual}\;\mathrm{fields}\;\mathrm{after}\;\mathrm{trypsinization}}{\mathrm{sum}\;\mathrm{of}\;\mathrm{number}\;\mathrm{of}\;\mathrm{cells}\kern0.24em \mathrm{in}\;\mathrm{five}\;\mathrm{visual}\;\mathrm{fields}\;\mathrm{before}\;\mathrm{trypsinization}}\times 100\;\% $$

The assays were performed in triplicate, and results are expressed as percentage mean ± SD. The protein contents of these cells were extracted for the detection of the adhesion molecule-integrin β2 (rabbit polyclonal antibody 1:500) (Santa Cruz Biotechnology, Cat. No. sc- 28661) by western blot assay.

### Morphology and cytoskeleton assay

#### F-actin labeling

To detect the actin cytoskeleton in microglia, Rhodamine Phalloidin (3.5:500, Cat. PHDR1, Cytoskeleton Inc., USA) was used. BV-2 microglial cells grown on poly-L-lysine coated cover slips were fixed with 4% paraformaldehyde for 10 min at room temperature. Cells were washed once with PBS at room temperature for 30 s and treated in permeabilization buffer (0.5% Triton X-100 in PBS) for 5 min at room temperature. After rinsing, the cells were incubated for 30 min in the dark at room temperature with Rhodamine Phalloidin (diluted in PBS according to the manufacturer’s specifications). Following incubation, the cells were rinsed in PBS and the cover slips were mounted onto glass slides with a fluorescent mounting medium.

#### Rho pathway

Rho GTPases are known to be involved in the control of actin cytoskeleton and cell migration. To investigate if scutellarin would affect the morphological changes of microglia *via* the Rho pathway, the protein supernatant of BV-2 microglial cells treated with 0.54 μM scutellarin was also extracted to detect for involvement of the Rho pathway using primary antibodies RhoA (mouse monoclonal IgG 1:500) (Santa Cruz Biotechnology, Cat. No. sc- 418) and Rac1 (mouse monoclonal IgG 1:1000) (Millipore Corporation, Cat. No. 2328346) by western blot assay.

#### Scanning electron microscopy (SEM)

For scanning electron microscopy (SEM), BV-2 microglial cells, control and treated with scutellarin, were seeded on cover slips in a 24-well plate at 2 × 10^4^/cm^2^. After prewashing three times with PBS, the cells were fixed in 2% paraformaldehyde and 3% glutaraldehyde in 0.1 M phosphate buffer at 4°C for 1 h before further fixation with 1% osmium tetroxide in PB, pH 7.4 for 30 min. Subsequently the samples were washed with 0.1 M PB and dehydrated through an ascending series of ethanol at room temperature before being transferred to a Bal*-*Tec CPD*-*030 critical point dryer (Bal*-*Tec *AG*, Balzers, Liechtenstein), using liquefied carbon dioxide as the transition fluid. The cover slips were then mounted on SEM stub. All cells were sputter-coated with 20 nm gold in a sputter coater (Balzers SCD 004) before examination in a scanning electron microscope (FEI 650 SEM).

#### Transmission electron microscopy (TEM)

Control and scutellarin treated BV-2 microglial cells were fixed in 2% paraformaldehyde and 3% glutaraldehyde in PB at 4°C for 1 h before post-fixation with 1% osmium tetroxide, pH 7.4 for 1 h. Subsequently, the cell samples were dehydrated through an ascending series of ethanol at room temperature before infiltration with acetone and resin followed by final embedding in resin which was allowed to polymerize at 60°C for 24 h. The cell samples were cut with an ultra-microtome (Leica). Ultrathin sections obtained were mounted on formvar-coated copper grids and double stained with uranyl acetate and lead citrate. The grids were viewed in a JEOL 1010 transmission electron microscope.

### Statistical analyses

SPSS 16.0 statistical software was used for statistical analysis. The data were expressed as mean ± SD and analyzed using one-way ANOVA followed by post-hoc analysis using Dunnet’s test to determine the statistical significance of different groups. All experiments were conducted in triplicate from different tissue samples. The difference was considered statistically significant when *P* <0.05.

## Results

### Scutellarin inhibited expression of NFκB in activated microglia

**Figure 1:**
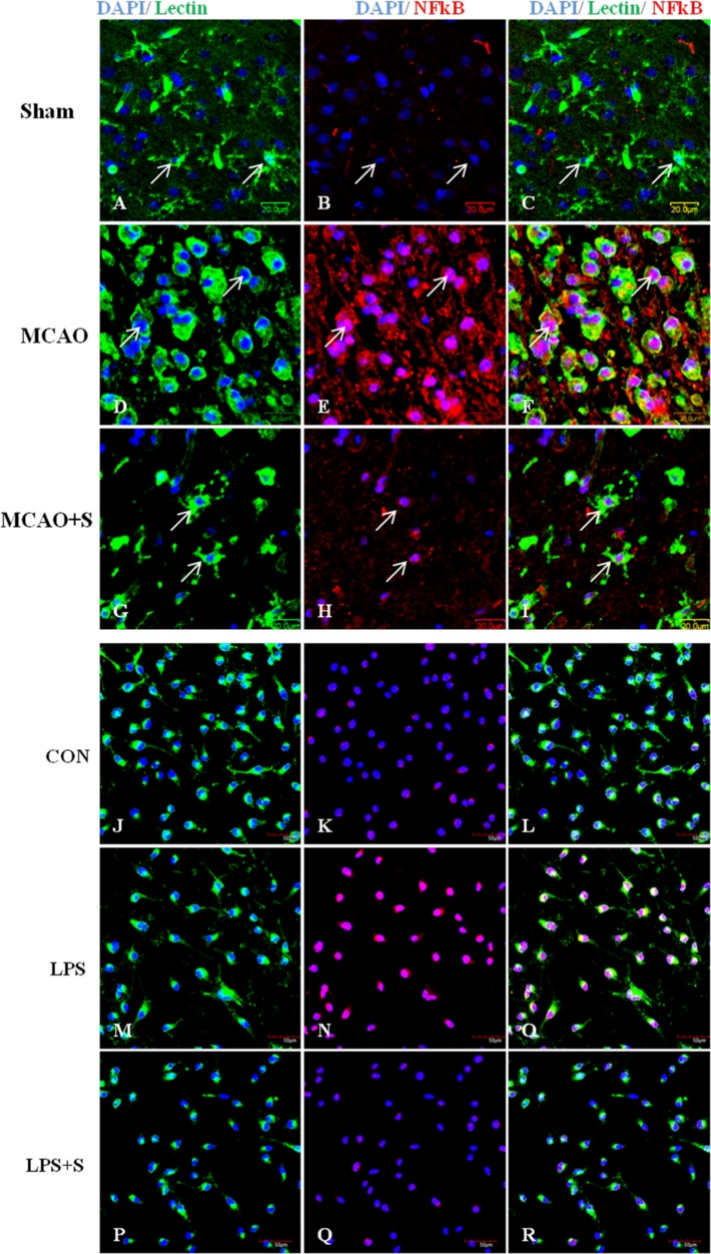
**Scutellarin reduced NF** **κ** **B expression in activated microglia** ***in vivo*** **and** ***in vitro*** **.** Confocal images showing the expression of nuclear factor kappa-light-chain-enhancer of activated B cells (NFκB) (red) in lectin + microglia (green, arrows) in the penumbral zones of MCAO rat **(D-F)** and following scutellarin treatment **(G-I)**. A drastic increase in the expression of NFκB **(E)** is evident in the activated microglia **(D)** in middle cerebral artery occlusion (MCAO) rat. Note that NFκB expression **(H**) is reduced in activated microglia **(G)** 7 days following treatment of MCAO rats with scutellarin. An upregulation of NFκB **(N)** is observed in lipopolysaccharide (LPS)-activated BV-2 microglia **(M)** in comparison to control **(J-L)**. NFκB is reduced **(Q)** in activated microglia treated with scutellarin. DAPI - blue. Scale bars in **A**-**I**: 20 μm, **J**-**R**: 50 μm.

NFκB expression in activated microglia was detected by double immunofluorescence staining in MCAO rats given scutellarin treatment. NFκB immunofluorescence in activated microglia in the penumbral zones was noticeably enhanced after MCAO, but it was evidently reduced at 7 days following treatment with scutellarin. Scutellarin also decreased the expression of NFκB in LPS-induced activated BV-2 microglia (Figure 1).

### Scutellarin reduced the expression of proteins in the Notch pathway in activated microglia in middle cerebral artery occlusion rats and in lipopolysaccharide-induced BV-2 microglia

**Figure 2:**
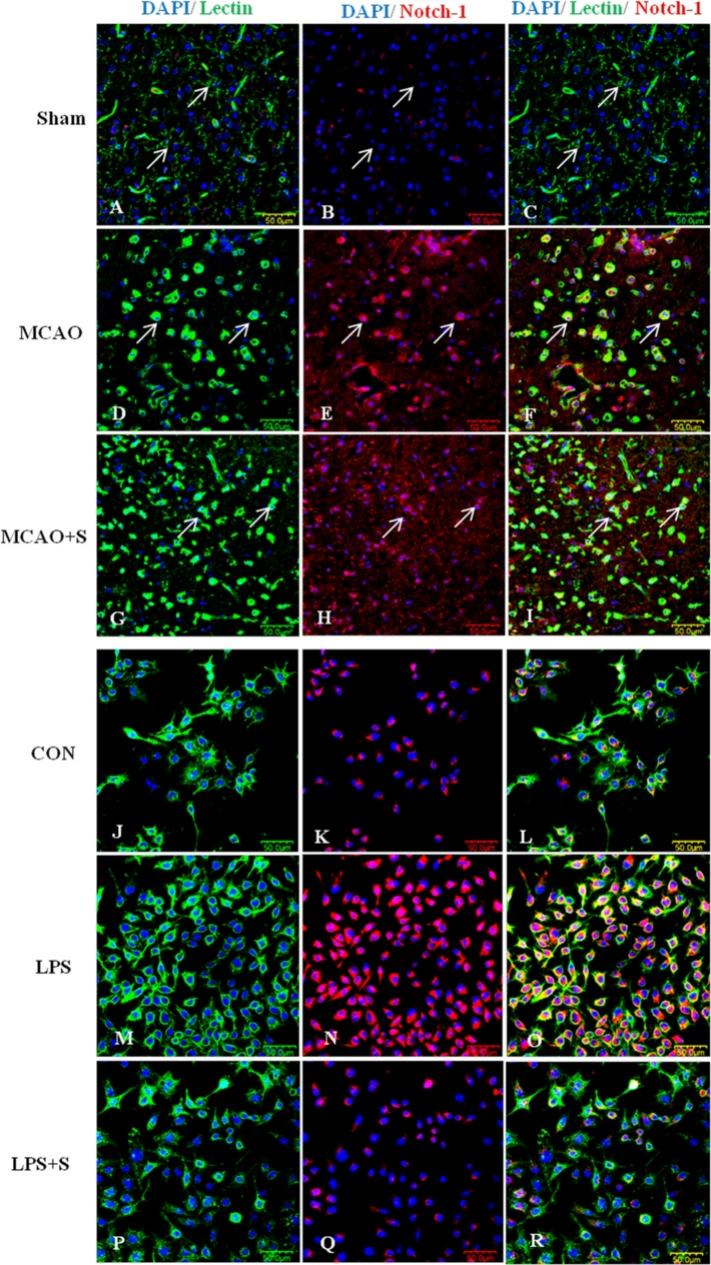
**Scutellarin reduced Notch-1 expression in activated microglia** ***in vivo*** **and** ***in vitro*** **.** Confocal images showing the expression of Notch-1 (red) in lectin + microglia (green, arrows) in the penumbral zones of middle cerebral artery occlusion (MCAO) rat **(D-F)** and following treatment with scutellarin **(G-I)**. An enhanced expression of Notch-1 **(E)** can be observed in the activated microglia **(D)** in MCAO rat. A noticeable reduction of Notch-1 expression **(H)** is observed in activated microglia **(G)** 7 days following treatment with scutellarin. An upregulation of Notch-1 **(N)** is observed in lipopolysaccharide (LPS)-activated BV-2 microglia **(M)** in comparison to control **(J-L)**. Notch-1 is reduced **(Q)** in activated microglia treated with scutellarin. DAPI - blue. Scale bars in **A**-**R**: 50 μm.

**Figure 3:**
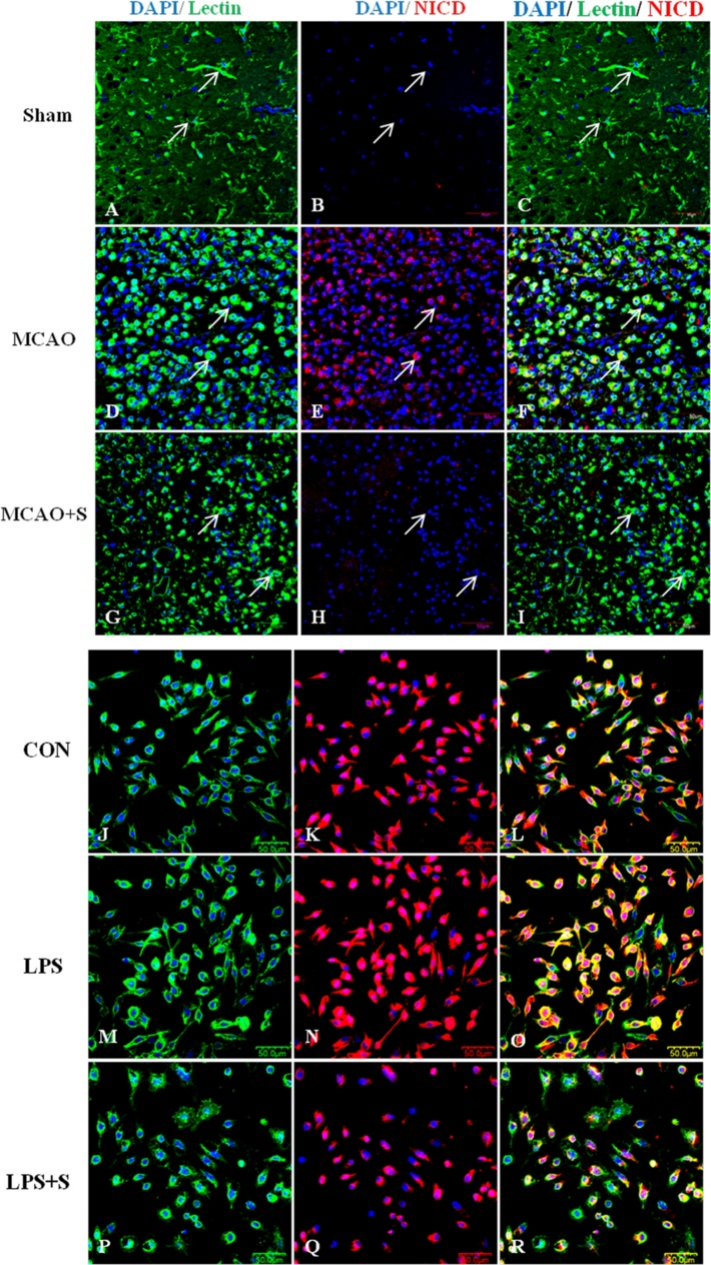
**Scutellarin reduced NICD expression in activated microglia** ***in vivo*** **and** ***in vitro*** **.** Confocal images showing the expression of Notch intracellular domain (NICD) (red) in lectin + microglia (green, arrows) in the penumbral zones of middle cerebral artery occlusion (MCAO) rat **(D-F)** and following treatment with scutellarin **(G-I)**. NICD expression **(E)** is augmented in the activated microglia **(D)**, but this is decreased **(H)** in activated microglia **(G)** 7 days following treatment with scutellarin. An upregulation of NICD **(N)** is observed in lipopolysaccharide (LPS)-activated BV-2 microglia **(M)** in comparison to control **(J-L)**. NICD is reduced **(Q)** in activated microglia treated with scutellarin. DAPI - blue. Scale bars in **A**-**R**: 50 μm.

**Figure 4:**
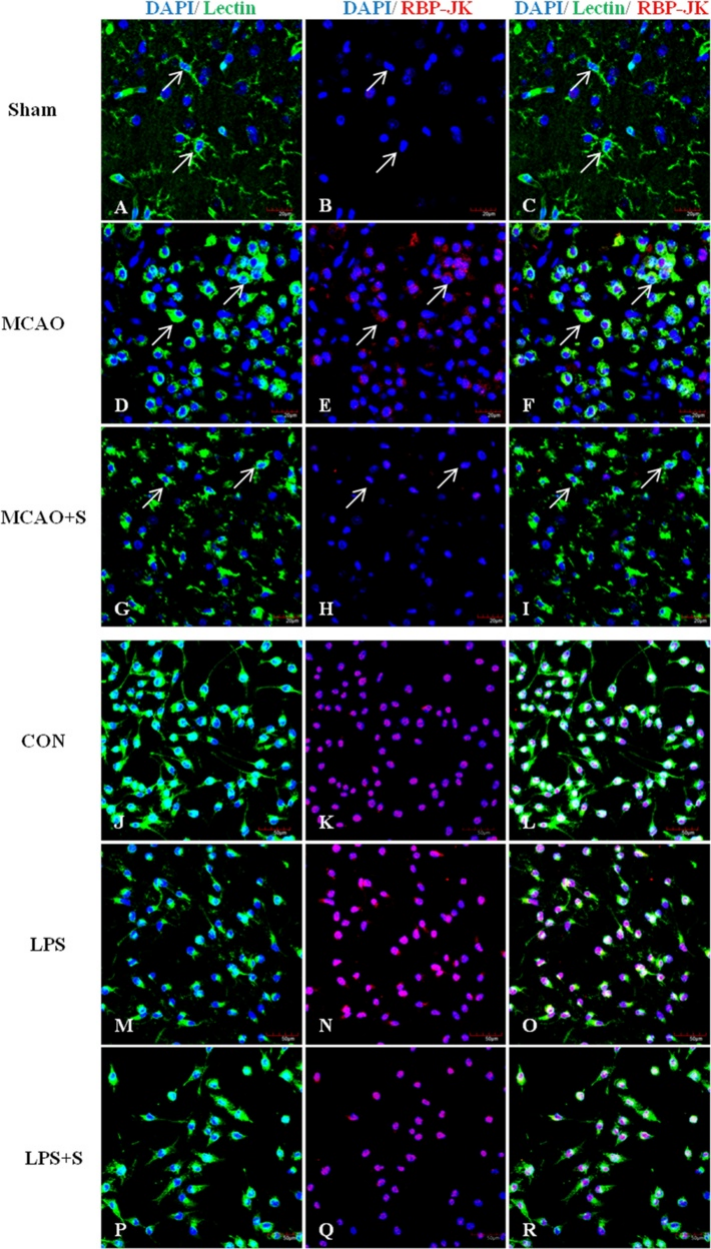
**Scutellarin reduced RBP-JK expression in activated microglia** ***in vivo*** **and** ***in vitro*** **.** Confocal images showing the expression of recombining binding protein suppressor of hairless (RBP-JK) (red) in lectin + microglia (green, arrows) in the penumbral zones of middle cerebral artery occlusion (MCAO) rat **(D-F)** and following treatment with scutellarin **(G-I)**. The expression of RBP-JK **(E)** is increased in the activated microglia **(D)** in MCAO rat. A noticeable reduction of RBP-JK expression **(H)** is observed in activated microglia **(G)** 7 days following treatment with scutellarin. An upregulation of RBP-JK **(N)** is observed in lipopolysaccharide (LPS)-activated BV-2 microglia **(M)** in comparison to control **(J-L)**. RBP-JK is decreased **(Q)** in activated microglia treated with scutellarin. DAPI - blue. Scale bars in **A**-**I**: 20 μm, **J**-**R**: 50 μm.

**Figure 5:**
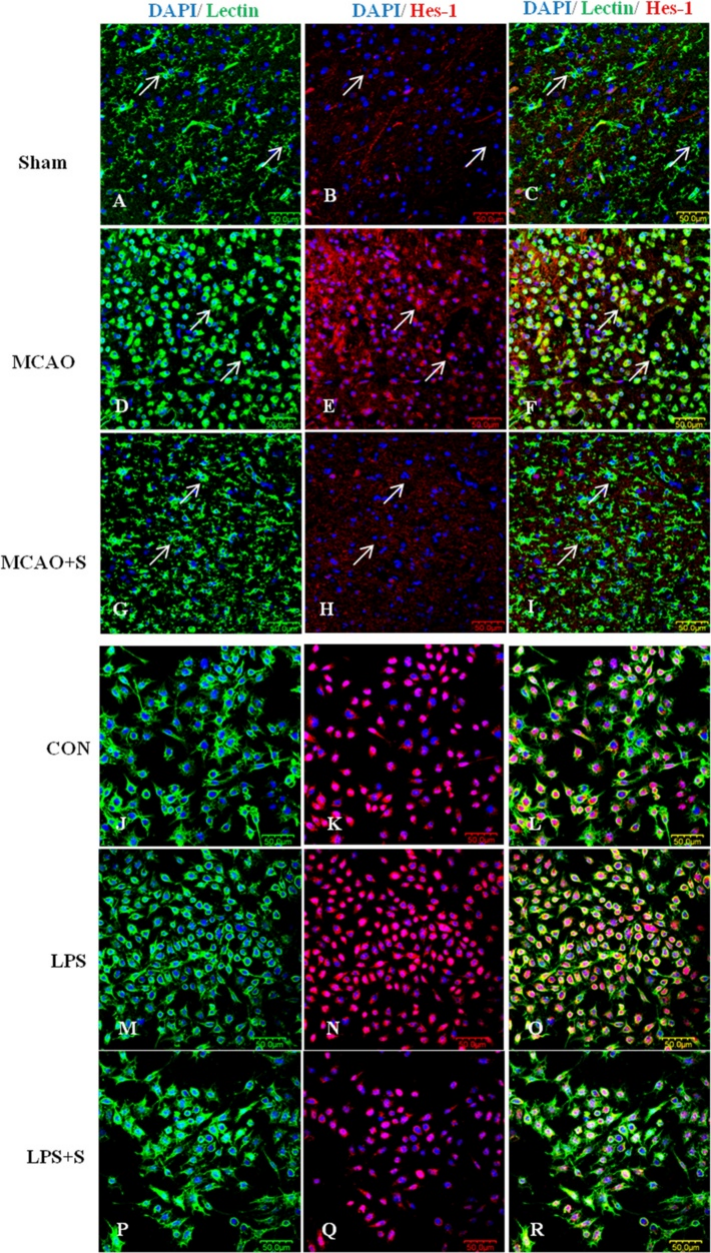
**Scutellarin reduced Hes-1 expression in activated microglia** ***in vivo*** **and** ***in vitro*** **.** Confocal images showing the expression of transcription factor hairy and enhancer of split-1 (Hes-1) (red) in lectin + microglia (green, arrows) in the penumbral zones of middle cerebral artery occlusion (MCAO) rat **(D-F)** and following treatment with scutellarin **(G-I)**. An increase in the expression of Hes-1 **(E)** is evident in the activated microglia **(D)** in MCAO rat. A noticeable reduction of Hes-1 expression **(H)** is observed in activated microglia **(G)** 7 days following treatment with scutellarin. An upregulation of Hes-1 **(N)** is observed in lipopolysaccharide (LPS)-activated BV-2 microglia **(M)** in comparison to control **(J-L)**. Hes-1 is reduced **(Q)** in activated microglia treated with scutellarin. DAPI - blue. Scale bars in **A**-**R**: 50 μm.

To investigate the effects of scutellarin on the Notch pathway, we examined the production of Notch-1, NICD, RBP-JK and Hes-1 in the brain tissue of MCAO rats given the treatment of low and high doses of the drug by double immunofluorescence staining, and in BV-2 microglia. Here we show the images of activated microglia in MCAO rats treated with a high dose of scutellarin only. Notch-1 immunofluorescence in activated microglia in the penumbral zones was noticeably enhanced after MCAO, but it was markedly reduced at 7 days following treatment with scutellarin (Figure 2). The expression of NICD (Figure 3), RBP-JK (Figure 4, Additional file 2: Figure S2) and Hes-1 (Figure 5, Additional file 3: Figure S3) paralleled with that of Notch-1 after treatment with scutellarin. On closer examination, RBP-JK and Hes-1 immunofluorescence was localized both in the cytoplasm and nucleus in activated microglia. The significance of this is considered in the discussion.

Consistent with results *in vivo*, expression changes in the Notch pathway proteins, including Notch-1, NICD, RBP-JK and Hes-1, were also observed in LPS-induced microglial BV-2 cells. Notch-1, NICD, RBP-JK and Hes-1 immunofluorescence intensity was markedly augmented when the cells were subjected to LPS treatment, but was reduced in LPS-activated microglia pretreated with scutellarin (Figures 2, 3, 4, and 5).

**Figure 6:**
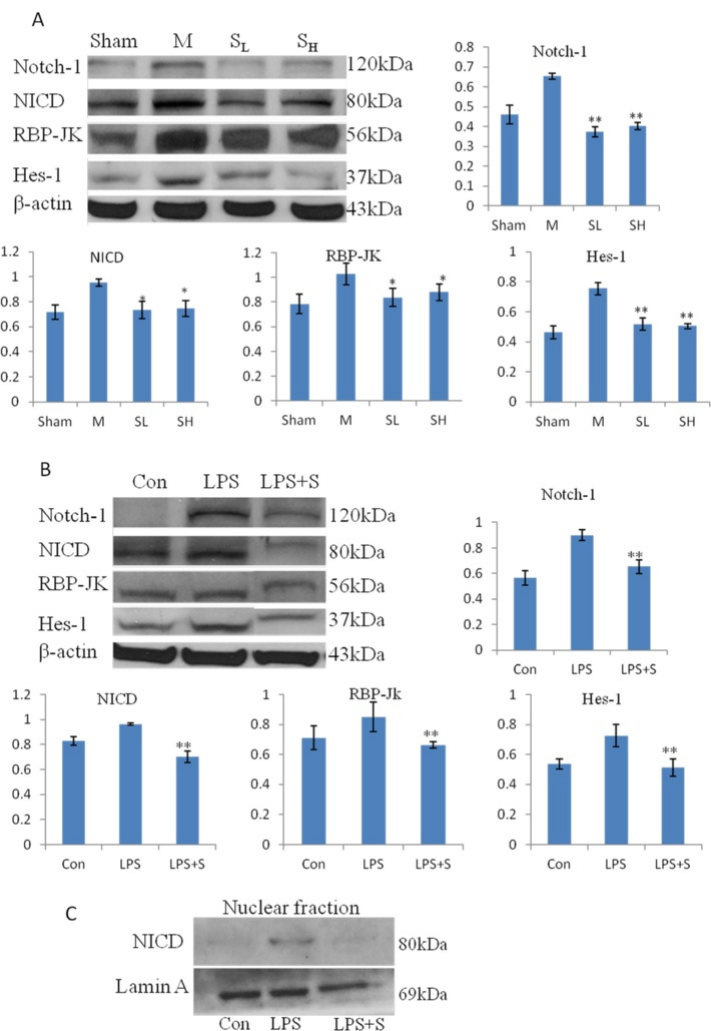
**Protein expression of Notch-1, NICD, RBP-JK and Hes-1 was decreased in MCAO rat and in BV-2 microglia following treatment with scutellarin. (A)** The expression levels of Notch-1, Notch intracellular domain (NICD), recombining binding protein suppressor of hairless (RBP-JK) and transcription factor hairy and enhancer of split-1 (Hes-1) in middle cerebral artery occlusion (MCAO) rat brains are depressed significantly at 7 days following treatment with scutellarin (low, S_L_ and high, S_H_ dose) when compared with the MCAO rats not treated with scutellarin. **(B)** The expression levels of Notch-1, NICD, RBP-JK and Hes-1 in lipopolysaccharide (LPS)-activated BV-2 microglial cells are reduced significantly when given scutellarin treatment. **(C)** Scutellarin decreases NICD translocation into the nucleus in lipopolysaccharide (LPS)-activated BV-2 microglial cells. Significant differences in protein levels are expressed as * *P* <0.05 and ** *P* <0.01. The values represent the mean ± SD in triplicate.

Western blot analysis also showed that the protein expression of Notch-1, NICD, RBP-JK and Hes-1 in the brain tissue of MCAO rats was obviously suppressed at 7 days after treatment with scutellarin (Figure 6A). There was, however, no obvious difference in the protein level treated with low and high dosage. The protein expression of Notch-1, NICD, RBP-JK and Hes-1 was also significantly decreased in BV-2 microglial cells pretreated with scutellarin (Figure 6B). The expression level of NICD in the nucleus, which was increased after LPS stimulation, was suppressed when pretreated with scutellarin (Figure 6C).

The protein expression of Notch-1, NICD, RBP-JK and Hes-1 in the brain tissue of MCAO rats was also augmented following ischemia and was also attenuated with scutellarin treatment at 1 and 3 days. There was no obvious difference in expression between different time points. Additional file 4: Figure S4 showed the protein expression by western blot and NICD immunofluroscence expression at 3 days.

### Scutellarin inhibited migration of microglia and reduced the expression of MCP-1 in activated microglia *in vivo* and *in vitro*

**Figure 7:**
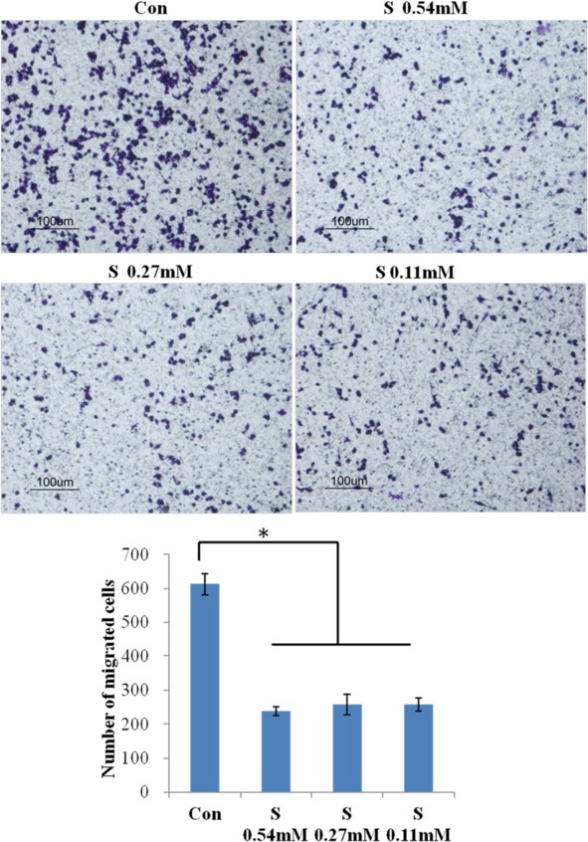
**Scutellarin inhibited migration of BV-2 microglia.** Transwell migration assay shows that scutellarin reduces the migration of BV-2 microglia. Light microscopy images of BV-2 cells treated with scutellarin of different concentrations and the control are shown. The quantitative analysis reveals a decrease in the migration of microglia with scutellarin treatment. Data are represented as mean ± SD (n = 3), * *P* <0.01. Scale bar = 100 μm.

Transwell migration experiment *in vitro* was performed to assess whether scutellarin influences the motility of microglia. Migration of BV-2 microglia towards the lower compartment treated with different concentrations of scutellarin was significantly decreased compared with the control; however, the effect on migration was not dose-dependent among groups (Figure 7).

**Figure 8:**
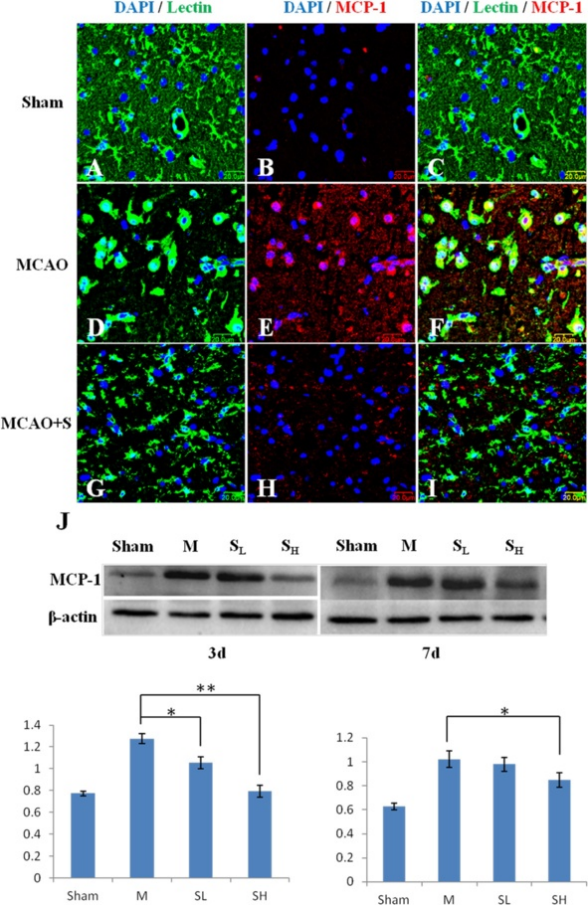
**Scutellarin reduced MCP-1 expression in activated microglia after middle cerebral artery occlusion (MCAO).** Confocal images showing the expression of monocyte chemoattractant protein-1 (MCP-1) (red) in lectin + microglia (green) in the penumbral zones of MCAO rat **(D-F)** and following treatment with Scutellarin **(G-I)**. Increase in MCP-1 expression **(E)** can be observed in the activated microglia **(D)** in MCAO rat. A marked reduction of MCP-1 expression **(H)** is observed in activated microglia **(G)** 7 days following treatment with scutellarin. DAPI - blue. Scale bars in **A**-**I**: 20 μm. The expression levels of MCP-1 in MCAO rat are depressed significantly at 3 and 7 days **(J)** following treatment with scutellarin at low (S_L_) and high (S_H_) dosage when compared with the MCAO rats not treated with the drug (compared with M). Significant differences in protein levels between MCAO and drug used rats are expressed as * *P* <0.01.

MCP-1 immunoexpression in activated microglia was detected by double immunofluorescence staining in MCAO rats given scutellarin treatment. MCP-1 immunofluorescence in activated microglia in the penumbral zones was noticeably enhanced after MCAO, but it was markedly reduced at 7 days following treatment with scutellarin. Western blot analysis also showed that the protein expression of MCP-1 was obviously suppressed at 3 and 7 days after treatment with the drug (Figure 8). It is noteworthy that at 7 days, there was no difference in MCP-1 expression between MCAO and scutellarin low dose (S_L_) group.

**Figure 9:**
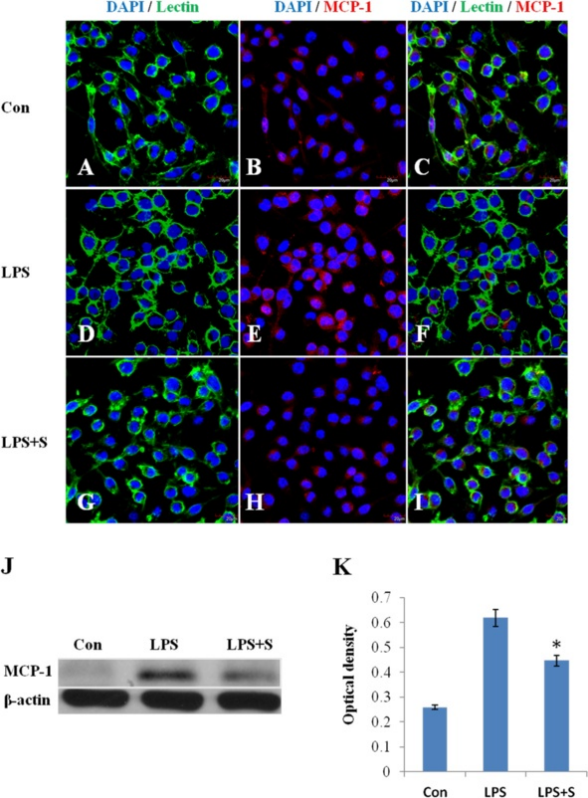
**MCP-1 expression was suppressed following treatment of lipopolysaccharide (LPS)-activated BV-2 microglia with scutellarin.** Confocal images show an upregulation of Monocyte chemoattractant protein-1 (MCP-1) **(E)** in LPS-activated BV-2 microglia **(D)** in comparison to control **(A-C)**. Pretreatment with scutellarin leads to decrease in the expression of MCP-1 **(H)** in LPS-activated BV-2 microglia **(G)**. DAPI - blue. Scale bars in **A**-**I**: 50 μm. The expression levels of MCP-1 in LPS-activated BV-2 microglial cells are depressed significantly following treatment with scutellarin by western blot **(J, K)**. Significant difference in protein level between LPS and scutellarin-treated cells are expressed as * *P* <0.01. The values represent the mean ± SD in triplicate.

As with results *in vivo*, expression change in MCP-1 was also observed in LPS-induced BV-2 microglial cells. MCP-1 immunofluorescence intensity was markedly augmented when the cells were subjected to LPS treatment, but was suppressed in LPS-activated microglia pretreated with scutellarin. The protein expression of MCP-1 was also significantly decreased in BV-2 microglial cells pretreated with the drug (Figure 9).

### Scutellarin increased the adhesion of BV-2 microglia and expression of integrin β2

**Figure 10:**
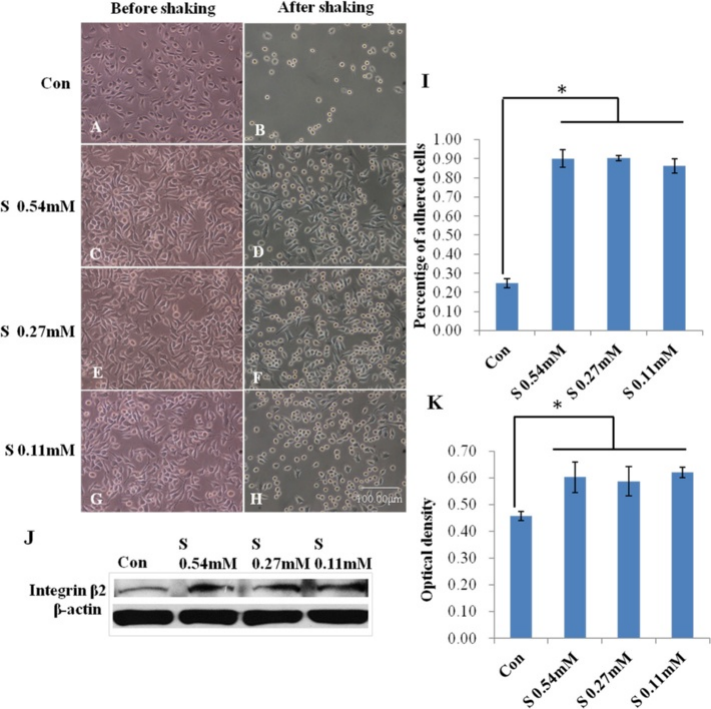
**Scutellarin promoted adhesion of BV-2 microglia.** Light microscope images show the number of cells in the control is markedly decreased **(B)** compared with that before trypsinization and shaking **(A)**. Treatment with scutellarin of different concentrations leads to an increase in numbers of attached cells **(D, F, H)**. The percentage of adherent cells treated with different concentrations of scutellarin significantly is increased as compared with the control **(I)**. Scale bars in **A**-**H**: 100 μm. The expression level of integrin β2 is upregulated following treatment with scutellarin of different concentrations **(J, K)**. Significant difference between control and scutellarin treated cells are expressed as * *P* <0.01. The values represent the mean ± SD in triplicate.

The cells tightly adhered to the wells in the control were markedly decreased in numbers after trypsinization and shaking, while the majority of activated BV-2 cells given Scutellarin for 3 h remained adherent to the wells. The frequency of adherent cells treated with different concentrations of scutellarin was significantly increased when compared with the control, but the change was not dose dependent (Figure 10A-I).

To further investigate the adhesive effects of scutellarin on microglia, we examined the production of integrin β2. The protein expression of integrin β2 was noticeably increased in BV-2 microglial cells treated with scutellarin of different concentrations. There was no significant difference between groups with the drug treatment (Figure 10J-K).

### Scutellarin alters the external morphology and ultrastructure of BV-2 microglial cells

#### F-actin labeling

**Figure 11:**
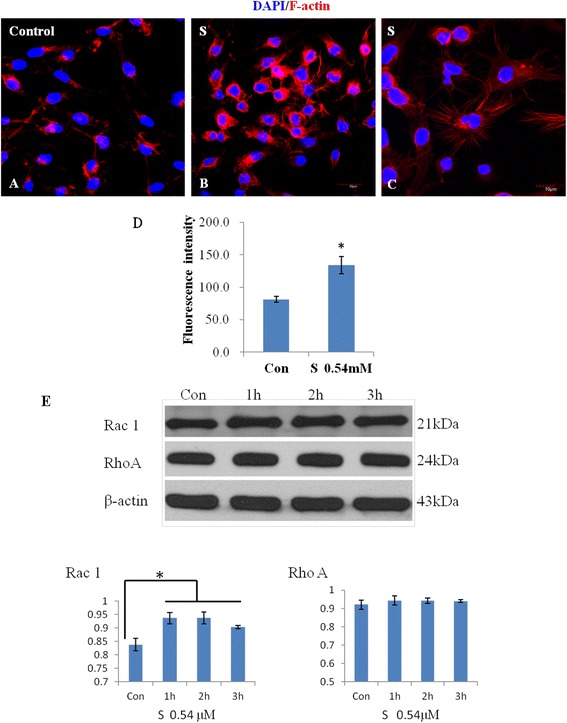
**Scutellarin increased the fluorescence intensity of F-actin, promoted the formation of projections and increased the expression of Rac1.** Confocal immunofluorescence images showing the expression of F-actin (Rhodamine, red) in BV-2 microglial cells. The expression of F-actin in the control cells **(A)** appears to be increased in cells treated with scutellarin. Note that microglia treated with scutellarin show more projections of actin microspikes **(B, C)**. Scale bar in **A**-**B**: 20 μm, **C**: 10 μm. The quantitative analysis shows that the intensity of F-actin in BV-2 microglial cells treated with scutellarin is increased compared with that of cells in control **(D)**. Data are presented as mean ± SD (n = 3), * *P* <0.01. The protein expression of Rac 1 is increased in BV-2 cells treated with scutellarin, but that of RhoA remains relatively unaltered **(E)**.

Actin bundles were orderly distributed in BV-2 microglia of the control, which shows a spherical outline (Figure 11A). BV-2 microglia treated with scutellarin exhibited more microspike projections and flattened or lamellar processes with intense staining of F-actin (Figure 11B, C). Scutellarin treatment promoted the formation of microspike projections and increase in actin staining intensity in BV-2 microglia (Figure 11D).

#### Rho pathway

The protein expression of Rac 1 was increased in BV-2 microglial cells treated with scutellarin from 1-3 h, but there was no significant difference among different time points. The expression level of RhoA was not altered by scutellarin (Figure 11E).

#### Electron microscopy

**Figure 12:**
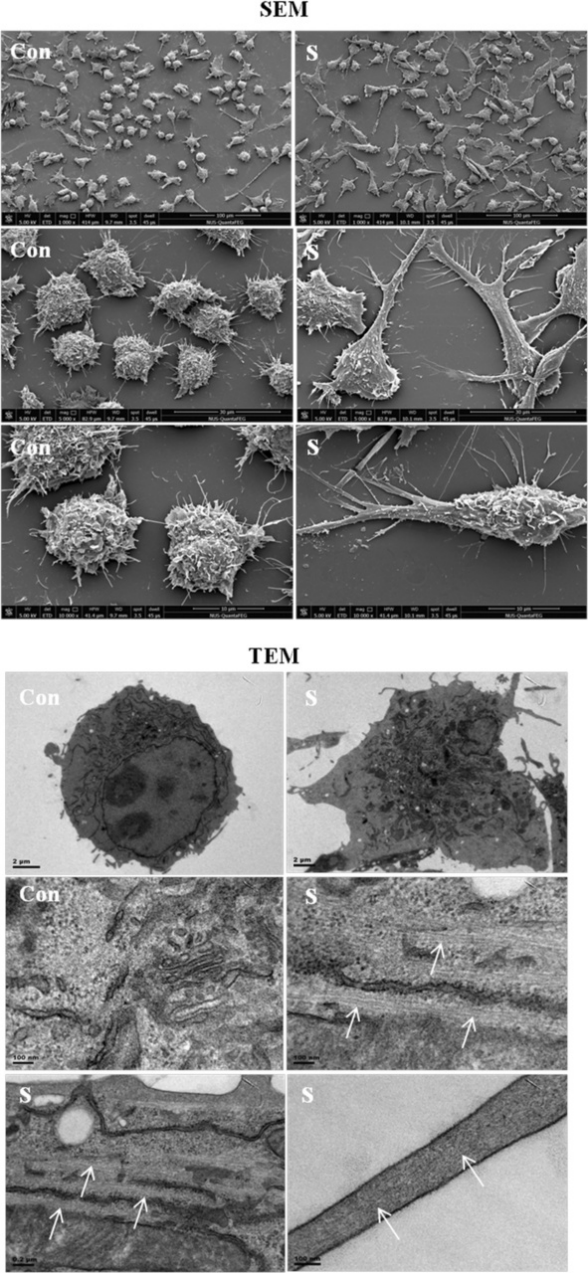
**Scutellarin treatment led to ultrastructural changes of BV-2 microglia.** Scanning electron microscopy (SEM) images show that the cells given scutellarin appear flattened with cell bodies that extend numerous processes emitting secondary and tertiary projections. Transmission electron microscopy (TEM) images show that the control BV-2 microglial cells have a relatively round cellular outline. They assume a more ramified phenotype when treated with scutellarin. Parallel arrays of microtubules/microfilaments (arrows) - both in the cell body and in the cytoplasmic processes - are observed after scutellarin treatment.

SEM images showed that the spherical BV-2 microglial cells in the control group emitted a few thin cytoplasmic processes. After scutellarin treatment, the cells appeared more flattened and the cell bodies extended numerous processes bearing secondary and tertiary projections. TEM images showed that the control cells exhibited a relatively round profile in section as compared with cells treated with scutellarin. In the latter, the cells were ramified. Bundles of microtubules and microfilaments in parallel arrays were distributed in the cell body and in broad and slender cytoplasmic processes after scutellarin treatment (Figure 12).

## Discussion

Neuroinflammation in the acute phase brain damage as well as chronic diseases has been reported to result in neuronal death, glia activation, synaptic impairment *etcetera*. The hallmark feature of neuroinflammation due to brain insult is the activation of microglia and microglia-mediated neuroinflammation has been extensively explored both *in vivo* with different experimental animal models in the developing and mature brain and *in vitro*. In this connection, it has been well documented that activated microglia releases a plethora of inflammatory mediators that may exacerbate the neurodegeneration process.

Microglial cells are responsible for the first line of immune defense in the CNS. In response to injury, microglia undergo progressive morphological and functional changes indicative of their activation. It has been well documented that microglia can scavenge cellular debris [23,24] and invading pathogens, as well as release neurotrophic factors that regulate the microenvironment stabilization [25]. However, over-activated microglia also release cytotoxic substances and proinflammatory cytokines which can aggravate injury of the tissues. Activation of microglia is thought to contribute to neuronal damage *via* the release of excessive proinflammatory cytokines and/or cytotoxic factors [26,27]. Therefore, suppressing over-activated microglia in pathological conditions seems to be an attractive therapeutic strategy for various neuroinflammatory or neurodegenerative disorders.

To date, many agents either synthetic or of natural sources have been documented to be able to block the inflammatory response driven by microglial activation [28-30]. Further progress has been made to identify active compounds extracted from herbal medicines which can modulate microglia-mediated inflammation. One of such compounds is scutellarin, the major active component extracted from *Erigeron breviscapus* (Vant.) Hand-Mazz [31]. It has been reported that scutellarin can inhibit LPS-induced production of proinflammatory mediators such as NO, TNF-α, IL-1β and ROS in rat primary microglia or BV-2 mouse microglial cell line [10,22].

NFκB is a transcription factor known to be one of the most important regulators of proinflammatory gene expression such as TNF-α, IL-1β, IL-6, IL-8, inducible nitric oxide synthase and cyclo-oxygenase-2 [32]. The Notch and NFκB pathways function synergistically in regulating several aspects of cellular functioning [33]. We have shown that the Notch pathway is upstream of NFκB and regulates activated microglia [14,15]. Previous studies have shown that scutellarin possesses the ability to suppress the expression of pro-inflammatory cytokine production in activated microglia, which is through the NFκB pathway [10,22]. Here, we confirm that scutellarin can inhibit the expression of NFκB in activated BV-2 microglia and activated microglia in MACO rats. In the present study, we aimed at ascertaining if scutellarin could disrupt the Notch pathway, thereby affecting NFκB-mediated cytokine production in activated microglia. Indeed, scutellarin suppressed the expression of members of the Notch signaling pathway such as Notch-1 and NICD, RBP-JK and Hes-1, as well as the nuclear translocation of NICD in activated microglia both *in vivo* and *in vitro*. A noteworthy feature in this study was the localization of RBP-JK and Hes-1 in activated microglia. It was noted that though they predominantly localize in the nucleus of activated microglia following translocation from the cytosol to the nucleus, some residual cytoplasmic localization and staining is also seen. Another possible explanation for this would be diffusion of these proteins from the nucleus during tissue section preparations. Notwithstanding, these results establish that scutellarin targets the Notch pathway, which lies upstream of NFκB [34].

Among the various cytokines released by activated microglia, MCP-1 plays an important role in the induction of cellular migration, blood-brain barrier alteration, and inflammation progress in the CNS [35]. MCP-1 can cause the migration and activation of microglia [36] inducing a series of inflammatory responses. It has been reported that scutellarin can inhibit cell migration [37,38]. Here we have shown that microglial migration was significantly inhibited by scutellarin with a corresponding reduction in the expression of MCP-1 in the microglia of MCAO rats and BV-2 microglial cells. Taken together, it is suggested that scutellarin exerts its inhibitory effect on microglial migration by reducing the expression of MCP-1.

Besides its effect on MCP-1, scutellarin increased the ability of adhesion in microglia which is consistent with its inhibitory effect on microglial migration. We also examined the expression of integrin β2 in microglia. β2 integrins play a major role in cell migration to the inflammatory lesion and also induce cytokine production [39]. It is known that β2 integrins as adhesive co-receptors are required for neutrophil responses triggered by a large number of stimuli such as TNF and other proinflammatory cytokines. We show here that the protein expression of the integrin β2 was noticeably increased in BV-2 microglial cells when treated with scutellarin. This provides strong morphological evidence that scutellarin can promote microglial adhesion by expressing integrin β2. It is therefore suggested that scutellarin can increase adhesion thereby decreasing migration of microglia.

An earlier report suggested that Hes-1, a downstream member of the Notch pathway, is a vital regulator of neuronal migration in the hypothalamus [40]. In our study, we found that scutellarin inhibited Hes-1 expression and microglia migration, but promoted microglia attachment. It is reasonable therefore to suggest that scutellarin can halt migration of activated microglia by inhibiting the Notch pathway.

BV-2 microglia underwent drastic morphological change following scutellarin treatment. By F-actin labeling, scutellarin was found to induce increased actin fluorescence intensity and altered the morphology of BV-2 microglia. BV-2 microglial cells given scutellarin treatment were highly ramified bearing many microspike projections and flattened processes when compared with the control. Furthermore, SEM images showed that when treated scutellarin, BV-2 microglial cells appeared to have a more flattened cell body with long extending primary processes that were flattened or lamellar and arising from these are many secondary projections. In contrast, the cells in the control group emitted thin processes. TEM images showed abundant microtubules/microfilaments both in the cell body and in cytoplasmic processes after scutellarin treatment. Taken together, it is justified to suggest that scutellarin affects the cytoskeletal arrangement or reorganization in microglial cells, therefore promoting the formation of flattened and microspike projections. Arising from the above, it is suggested that scutellarin inhibits the migration of microglia and this may be attributed to its effects on the reorganization and stabilization of cytoskeletal dynamics.

To understand how scutellarin would exert its effect on the cytoskeletal formation, we detected the expression of Rho family small GTPases (Rho GTPases). Rho GTPases are involved in the control of actin cytoskeleton and cell migration. RhoA and Rac1 are members of Rho GTPases [41]. Rac1 stimulates actin polymerization and Rac1 activation induces formation of lamellae of cells and cell spreading on extracellular matrixes. Here, we found the expression of Rac1 was increased in BV-2 microglial cells after scutellarin treatment, but RhoA expression was not affected. In view of this, it is suggested that Rac1 may play an important role in promoting extension of cytoskeleton and formation of flattened processes in scutellarin treated microglia.

Finally, contrary to the reports by others in which the Notch pathway has been implicated in migration and phagocytosis in different cells, scutellarin did not appear to exert an obvious effect on microglial migration, adhesion and phagocytosis *via* the Notch pathway as shown by the DAPT (a γ-secretase inhibitor) treatment, and phagocytosis of latex beads (YY, PR and E-A L, unpublished data). This suggests that while scutellarin can directly affect the Notch pathway, the effects on microglial behaviors seem to involve other pathways but this remains to be further explored.

## Conclusion

It is unequivocal from this study that scutellarin can attenuate the NFκB expression and inhibit the nuclear translocation of NFκB in activated microglia. Coupled with this, scutellarin inhibits the Notch pathway by reducing the expression of Notch-1, NICD, RBP-JK and Hes-1 in microglia *in vivo and in vitro*. Scutellarin increased the ability of adhesion in microglia, while it inhibited the expression of MCP-1 and microglial migration. It also changed the phenotype of microglia by promoting the formation of processes and rearranging the cytoskeleton. The present results have provided the first morphological evidence that scutellarin regulated the activation of microglia *via* the Notch pathway and induced the morphological and functional changes in activated microglia. This may be effected *via* the NFκB pathway, which is known to operate synergistically with the Notch pathway. Other pathways, however, should also be considered but this awaits further investigation. Notwithstanding, the present results have provided strong experimental evidence supporting scutellarin as a therapeutic strategy to attenuate microglia-mediated neuroinflammation in ischemic/hypoxic brain injury.

## Additional files

Additional file 1: Figure S1. Scutellarin decreased the protein expression of iNOS, TNF-α and Notch-1 in a dose-dependent manner in activated BV-2 microglia. BV-2 cells were pretreated with Scutellarin at 0.02, 0.1, and 0.54 mM for 1 h and then stimulated with lipopolysaccharide (LPS) 1 μg/ml for 3 h. The expression levels of inducible nitric oxide synthase (iNOS), tumor necrosis factor-alpha (TNF-α) and Notch-1 in LPS-activated BV-2 microglial cells are reduced significantly being most drastic with scutellarin treatment at 0.54 mM. Significant differences in protein levels are expressed as * *P* <0.05 and ** *P* <0.01. The values represent the mean ± SD in triplicate. Additional file 2: Figure S2. Scutellarin reduced RBP-JK expression in activated microglia *in vivo*. Confocal images showing the expression and nuclear localization of recombining binding protein suppressor of hairless (RBP-JK) (red) in lectin + microglia (green, arrows) in the penumbral zones of middle cerebral artery occlusion (MCAO) rat (D-F) and following treatment with scutellarin (G-I). RBP-JK expression (E) was markedly increased and localized to the nucleus in activated microglia (D) in MCAO rat. Arrows in E and H show nuclear localization of RBP-JK. At 7 days following treatment of MCAO rats with scutellarin, RBP-JK expression (H) became hardly detectable in microglia (G). DAPI – blue. Scale bars in A-I: 20 μm. Additional file 3: Figure S3. Scutellarin-reduced Hes-1 expression in activated microglia *in vivo*. Confocal images showing the expression and nuclear localization of transcription factor hairy and enhancer of split-1 (Hes-1) (red) in lectin + microglia (green, arrows) in the penumbral zones of middle cerebral artery occlusion (MCAO) rat (D-F) and following treatment with scutellarin (G-I). Hes-1 expression (E) was markedly increased and localized to the nucleus in activated microglia (D) in MCAO rat. Arrows in E and H show nuclear localization of Hes-1. At 7 days following treatment of MCAO rats with scutellarin, Hes-1 expression (H) was attenuated in microglia (G). DAPI – blue. Scale bars in A-I: 20 μm. Additional file 4: Figure S4. Scutellarin-decreased NICD immunofluorescence and protein expression of Notch-1, NICD, RBP-JK and Hes-1 in middle cerebral artery occlusion (MCAO) rats 3 days following treatment with scutellarin. Confocal images showing the expression of Notch intracellular domain (NICD) (red) in lectin + microglia (green, arrows) in the penumbral zones of MCAO rat (D-F) and following treatment with scutellarin (G-I). NICD expression (E) is increased in the activated microglia (D), but is decreased (H) in activated microglia (G) 3 days following treatment with scutellarin. The expression levels of Notch-1, NICD, recombining binding protein suppressor of hairless (RBP-JK) and transcription factor hairy and enhancer of split-1 (Hes-1) in MCAO rat brains is reduced significantly at 3 days following treatment with scutellarin (S_L_ and S_H_ dose) when compared with MCAO rats not treated with scutellarin. Significant differences in protein levels are expressed as * *P* <0.05 and ** *P* <0.01. The values represent the mean ± SD in triplicate.

## Abbreviations

CNS: central nervous system
Hes-1: transcription factor hairy and enhancer of split-1
IL-1β: interleukin-1 beta
iNOS: inducible nitric oxide synthase
LPS: lipopolysaccharide
MCAO: middle cerebral artery microglia
MCP-1: monocyte chemoattractant protein-1
NF-κB: nuclear factor kappa-light-chain-enhancer of activated B cells
NICD: Notch intracellular domain
NO: nitric oxide
RBP-JK: recombining binding protein suppressor of hairless
Rho GTPases: Rho family small GTPases
ROS: reactive oxygen species
SEM: scanning electron microscopy
S_H_: scutellarin high dose
S_L_: scutellarin low dose
TEM: transmission electron microscopy
TNF-α: tumor necrosis factor-alpha
