# Supplementary figures and images for: Scutellarin regulates the Notch pathway and affects the migration and morphological transformation of activated microglia in experimentally induced cerebral ischemia in rats and in activated BV-2 microglia
Source: J Neuroinflammation. 2015 Jan 20;12:11. doi: 10.1186/s12974-014-0226-z (PMC4316603; doi:10.1186/s12974-014-0226-z)

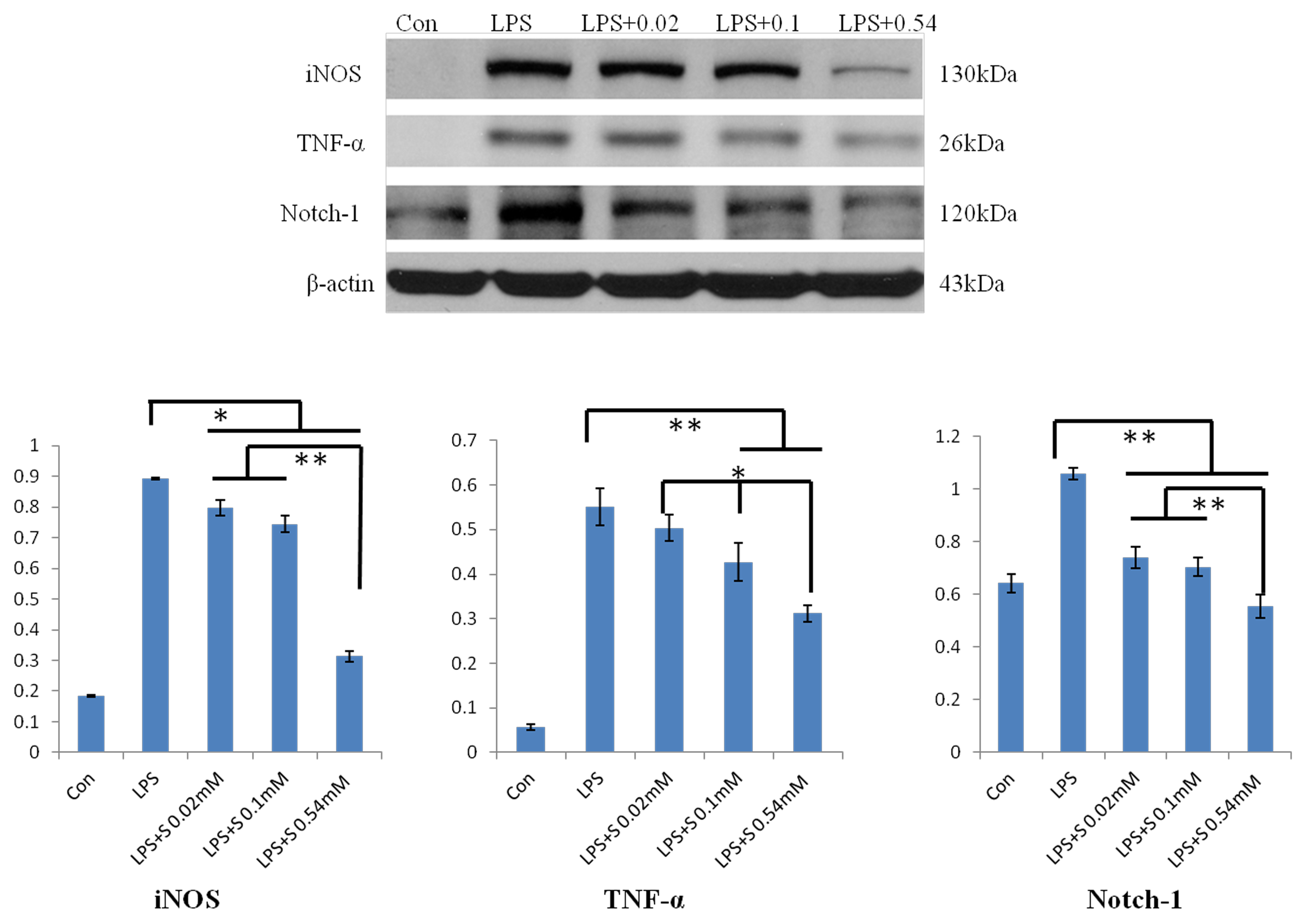

Supplement: Additional file 1: Figure S1. — Scutellarin decreased the protein expression of iNOS, TNF-α and Notch-1 in a dose-dependent manner in activated BV-2 microglia. BV-2 cells were pretreated with Scutellarin at 0.02, 0.1, and 0.54 mM for 1 h and then stimulated with lipopolysaccharide (LPS) 1 μg/ml for 3 h. The expression levels of inducible nitric oxide synthase (iNOS), tumor necrosis factor-alpha (TNF-α) and Notch-1 in LPS-activated BV-2 microglial cells are reduced significantly being most drastic with scutellarin treatment at 0.54 mM. Significant differences in protein levels are expressed as * P <0.05 and ** P <0.01. The values represent the mean ± SD in triplicate. [file 12974_2014_226_MOESM1_ESM.tiff]

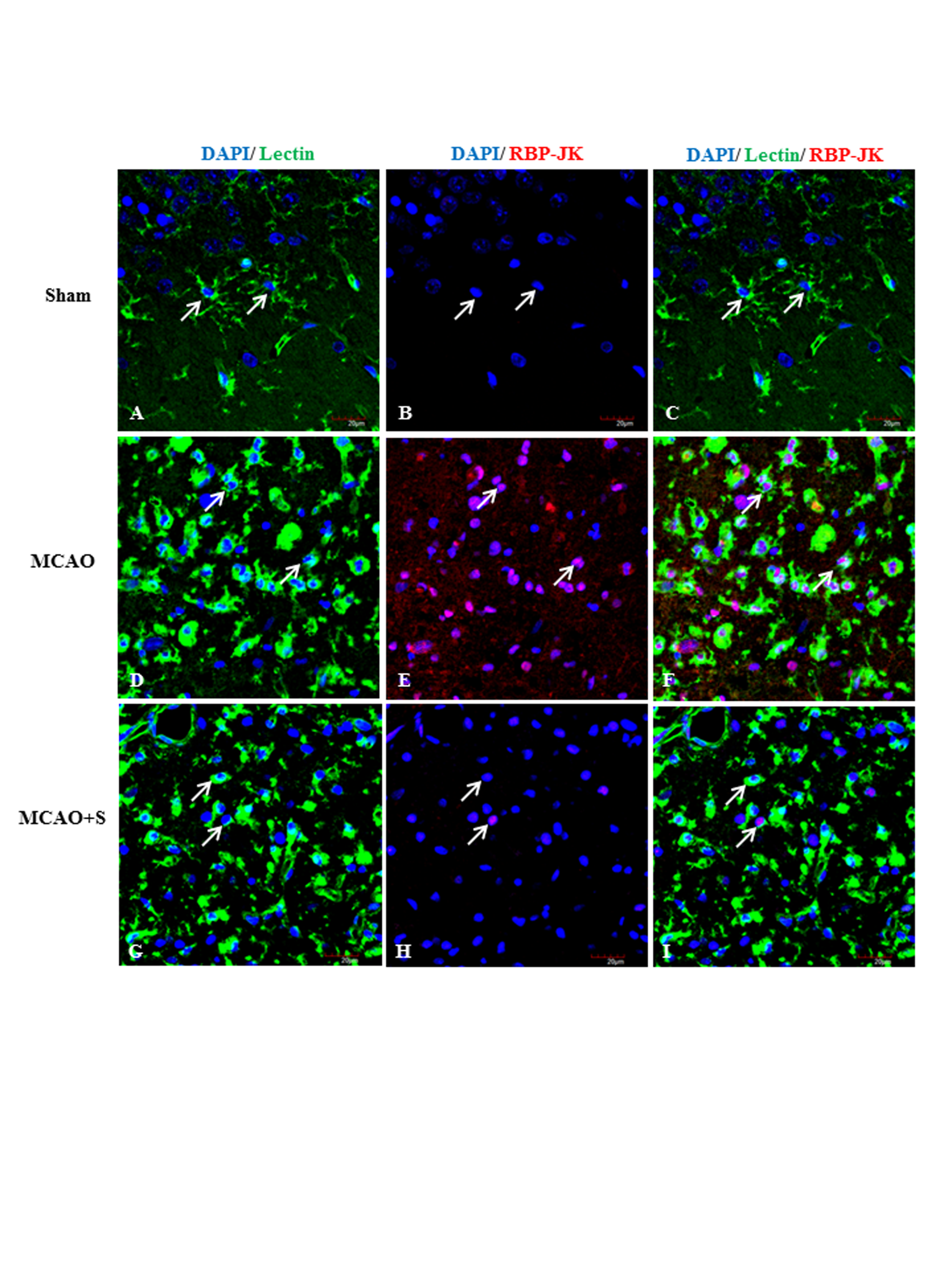

Supplement: Additional file 2: Figure S2. — Scutellarin reduced RBP-JK expression in activated microglia in vivo. Confocal images showing the expression and nuclear localization of recombining binding protein suppressor of hairless (RBP-JK) (red) in lectin + microglia (green, arrows) in the penumbral zones of middle cerebral artery occlusion (MCAO) rat (D-F) and following treatment with scutellarin (G-I). RBP-JK expression (E) was markedly increased and localized to the nucleus in activated microglia (D) in MCAO rat. Arrows in E and H show nuclear localization of RBP-JK. At 7 days following treatment of MCAO rats with scutellarin, RBP-JK expression (H) became hardly detectable in microglia (G). DAPI – blue. Scale bars in A-I: 20 μm. [file 12974_2014_226_MOESM2_ESM.tiff]

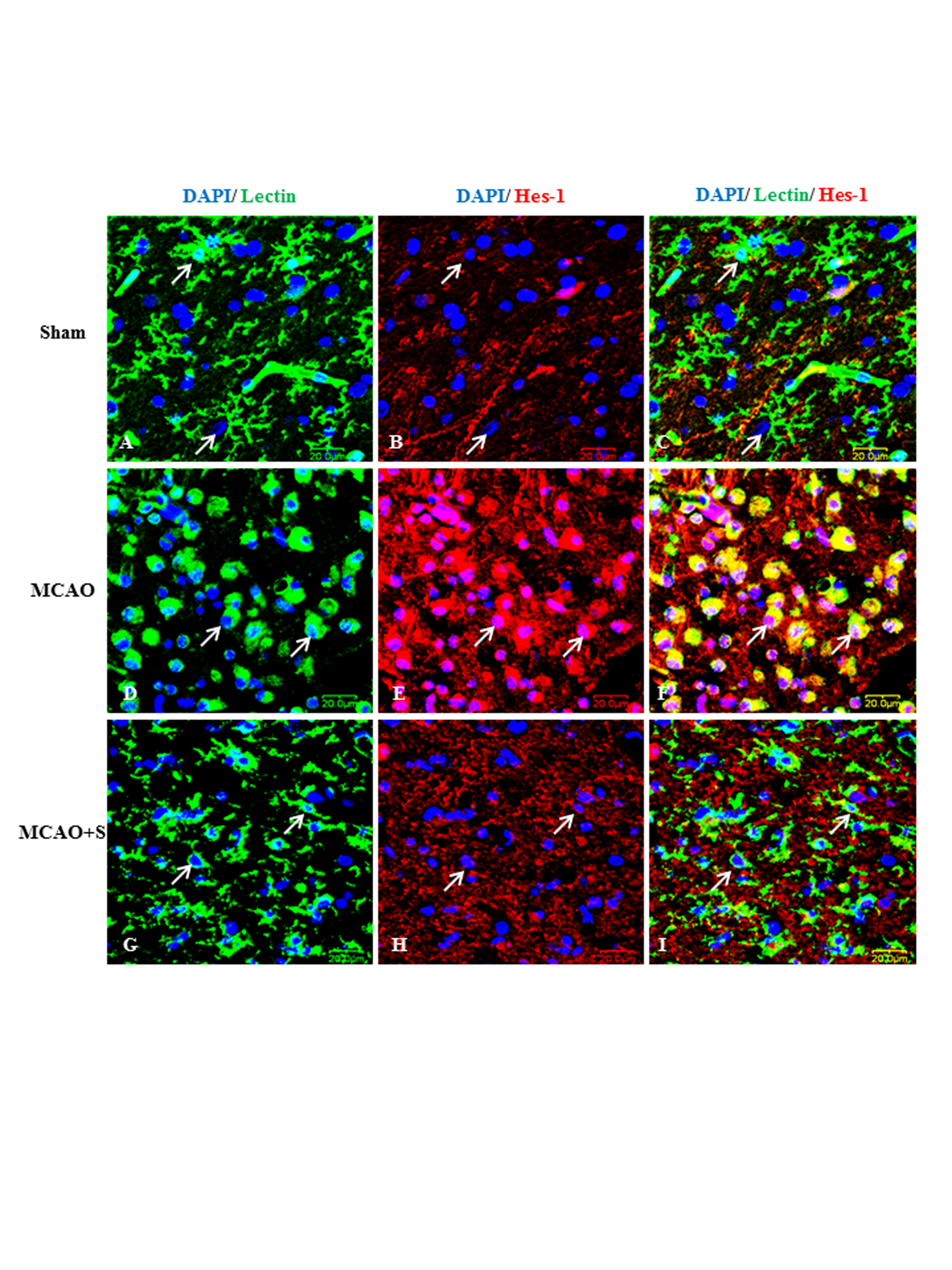

Supplement: Additional file 3: Figure S3. — Scutellarin-reduced Hes-1 expression in activated microglia in vivo. Confocal images showing the expression and nuclear localization of transcription factor hairy and enhancer of split-1 (Hes-1) (red) in lectin + microglia (green, arrows) in the penumbral zones of middle cerebral artery occlusion (MCAO) rat (D-F) and following treatment with scutellarin (G-I). Hes-1 expression (E) was markedly increased and localized to the nucleus in activated microglia (D) in MCAO rat. Arrows in E and H show nuclear localization of Hes-1. At 7 days following treatment of MCAO rats with scutellarin, Hes-1 expression (H) was attenuated in microglia (G). DAPI – blue. Scale bars in A-I: 20 μm. [file 12974_2014_226_MOESM3_ESM.tiff]

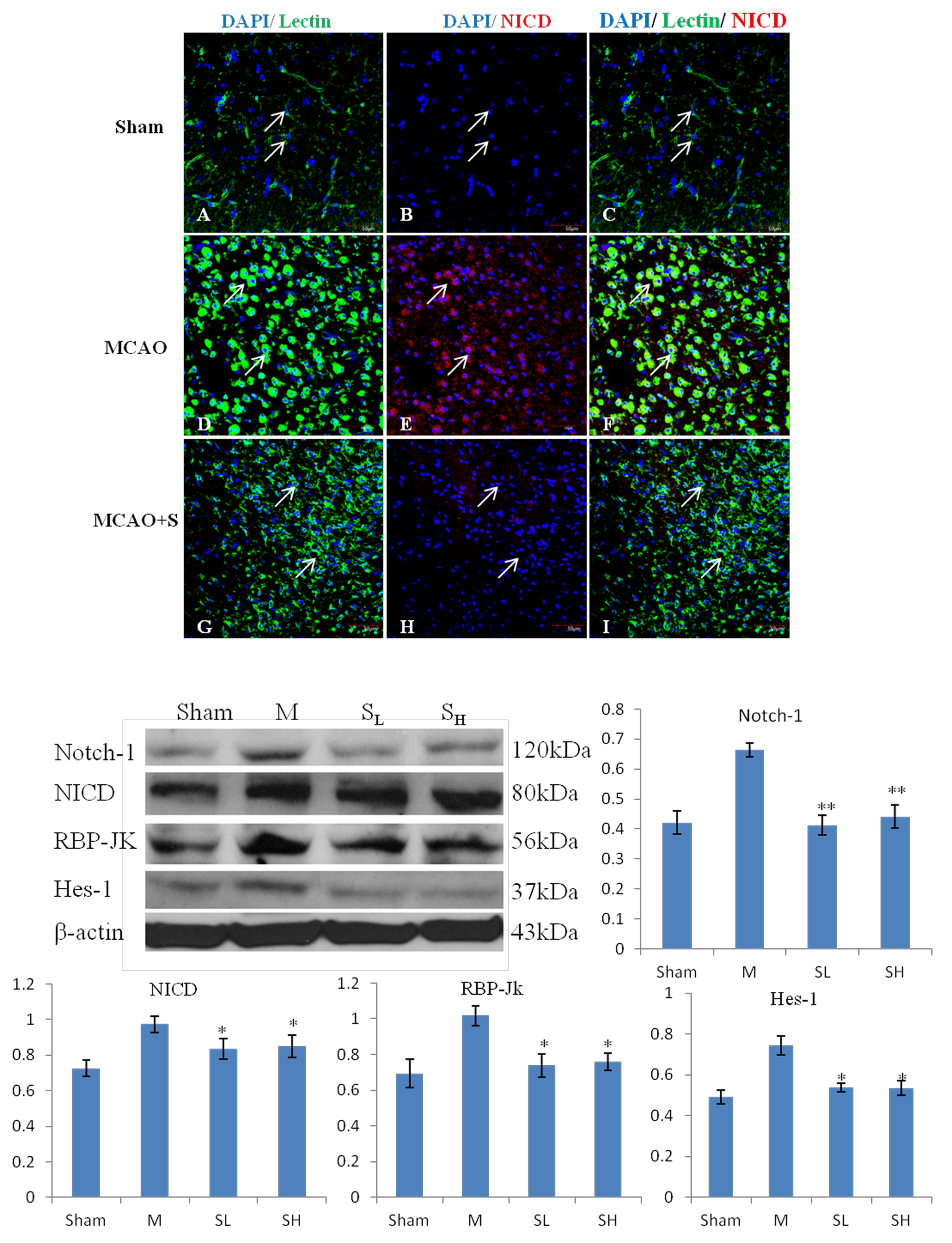

Supplement: Additional file 4: Figure S4. — Scutellarin-decreased NICD immunofluorescence and protein expression of Notch-1, NICD, RBP-JK and Hes-1 in middle cerebral artery occlusion (MCAO) rats 3 days following treatment with scutellarin. Confocal images showing the expression of Notch intracellular domain (NICD) (red) in lectin + microglia (green, arrows) in the penumbral zones of MCAO rat (D-F) and following treatment with scutellarin (G-I). NICD expression (E) is increased in the activated microglia (D), but is decreased (H) in activated microglia (G) 3 days following treatment with scutellarin. The expression levels of Notch-1, NICD, recombining binding protein suppressor of hairless (RBP-JK) and transcription factor hairy and enhancer of split-1 (Hes-1) in MCAO rat brains is reduced significantly at 3 days following treatment with scutellarin (SL and SH dose) when compared with MCAO rats not treated with scutellarin. Significant differences in protein levels are expressed as * P <0.05 and ** P <0.01. The values represent the mean ± SD in triplicate. [file 12974_2014_226_MOESM4_ESM.tiff]
